# Virtual Morphometry of the First Lumbar Vertebrae for Estimation of Sex Using Computed Tomography Data in the Turkish Population

**DOI:** 10.7759/cureus.16597

**Published:** 2021-07-23

**Authors:** Mustafa Bozdag, Gokce Karaman

**Affiliations:** 1 Radiology, Tepecik Training and Research Hospital, Izmir, TUR; 2 Department of Forensic Science, Council of Forensic Medicine, Manisa, TUR

**Keywords:** lumbar, morphometry, tomography, vertebrae, turkish

## Abstract

Introduction

It may be necessary to make sex estimation by examining other bones that have been obtained intact. Vertebrae, especially the thoracic and lumbar vertebrae, are among the best-preserved skeletal elements from the forensic and archaeological point of view. Therefore, lumbar vertebrae can become an important skeletal element for sex estimation. In our study, measurements were made on the CT images of the first lumbar vertebra, and the accuracy of sex estimation from the L1 vertebra was investigated in the Turkish population.

Materials and methods

Three dimensional (3D) models of the L1 vertebra were created from CT images of 241 (121 females and 120 males) individuals. Twenty-two linear measurements were taken in lateral, anterior, and superior views of 3D models of the L1 vertebra. Univariate and multivariate discriminant function analyzes were applied to the measured parameters to determine predictive rates of sex. Intra- and interobserver errors were calculated.

Results

All linear measurements were higher in males than females. All parameters excluding SCD (Spinal canal depth), PLu (Upper pedicle length), PLI (Lower pedicle length), AHi (Inferior articular process height), and SPH (Spinous process height) showed statistically significant differences between sex. The highest rate of 70.5% was obtained for the EPWu (Upper end-plate width) and EPWl (Lower end-plate width) parameters. When all variables of L1 vertebra were included in the stepwise discriminant analysis, correct prediction rates were determined as 72.6%.

Conclusion

Our study is the first study in which L1 vertebrae are examined with the purpose of sex estimation in the Turkish population and we think that our data will be an important reference for sex estimation from the L1 vertebra in the Turkish population.

## Introduction

Identification of skeletal, severely degraded, or unidentified human remains is important for forensic and humanitarian reasons [1-3]. In the identification process; age, sex, stature, and other unique features of the person are determined. Since other analyses are sex-dependent methods, sex estimation is usually the first step in the biological identification process [4-5].

Although the most reliable results in sex estimation with morphological and metric methods are obtained from the analyses of the hip and skull bones; in cases such as natural disasters and mass disasters, it may not always be possible to examine the skull and hip bones due to animal activities, voluntary damage and deterioration of the skeleton due to taphonomic processes [2-3]. It may be necessary to estimate sex by examining any intact bones that have been obtained. For this reason, it is necessary to develop different methods for sex estimation with bones other than hip and skull bones. Today, many parts of the adult skeleton are used for sex estimation [3]. Studies on the vertebral column have also shown that the vertebrae demonstrate sexual dimorphic features [6-15].

Lumbar vertebrae are the largest of the vertebrae and usually consist of five vertebrae. They have a larger vertebral body due to their load-bearing properties [16]. They have no costal facets and transverse foramina. The vertebral foramen is triangular in shape and is larger compared to the thoracic vertebrae, but smaller than the cervical vertebrae [16]. Vertebrae, especially the thoracic and lumbar vertebrae, are among the best-preserved skeletal elements from the forensic and archaeological point of view [17-19]. Therefore, lumbar vertebrae can become an important skeletal element for sex estimation.

 Zheng et al. stated that sex estimation can be made with an accuracy of 88.6% using discriminant function analysis from the L1 vertebra [11]. Ostrofsky and Churchill also stated that among the lumbar vertebrae, the L1 was the most sex diagnostic vertebra with an accuracy rate of 87.1% for sex estimation [12]. Decker et al. reported that each of the lumbar vertebrae demonstrated sexual dimorphism exceeding 80% using discriminant function analysis [14]. Azofra-Monge and Alemán Aguilera in their study evaluating the lumbar vertebrae stated that the lumbar vertebrae showed more sexual dimorphism in their study compared to previous studies, and the highest accuracy rates in the regression equations were obtained from the L1 vertebra (90.1-94.5%) [15]. Studies show that sex estimation can be made using lumbar vertebrae with high accuracy rates [11-12,14-15]. It has also been shown in studies that, of the five lumbar vertebrae, especially the first lumbar vertebra is more sexually dimorphic than the other lumbar vertebrae [12,14].

The degree of sexual dimorphism present in postcranial skeletal elements may differ between populations, therefore, the most accurate sex marker may differ between populations [20]. It is stated that vertebral discriminant function analyzes used in sex estimation are also population-specific [17]. For this reason, when sex estimation is made using vertebrae, employing data obtained from studies conducted on that particular population will increase the accuracy of sex estimation.

There is no study in the Turkish population that evaluated the lumbar vertebrae in terms of sex predicting accuracy. In our study; lumbar computed tomography (CT) images were examined, measurements were made on the first lumbar vertebra, and the accuracy of sex estimation from L1 vertebra in the Turkish population was investigated by applying discriminant function analysis.

## Materials and methods

This retrospective study was approved by the Hospital Research Ethics Committee (protocol number: 2020/11-29) and the study was conducted in accordance with the standards of the Declaration of Helsinki. This study was conducted on 241 people (121 females and 120 males) from the west of Turkey. These individuals were patients who underwent CT scans without contrast medium because of urinary stone diseases from January 2016 to December 2020. First lumbar vertebra CT images of individuals were retrospectively evaluated. Cases that had any bone pathologies, degenerative bone diseases, fractured or deformed L1 vertebra were excluded from the study.

**Figure 1:**
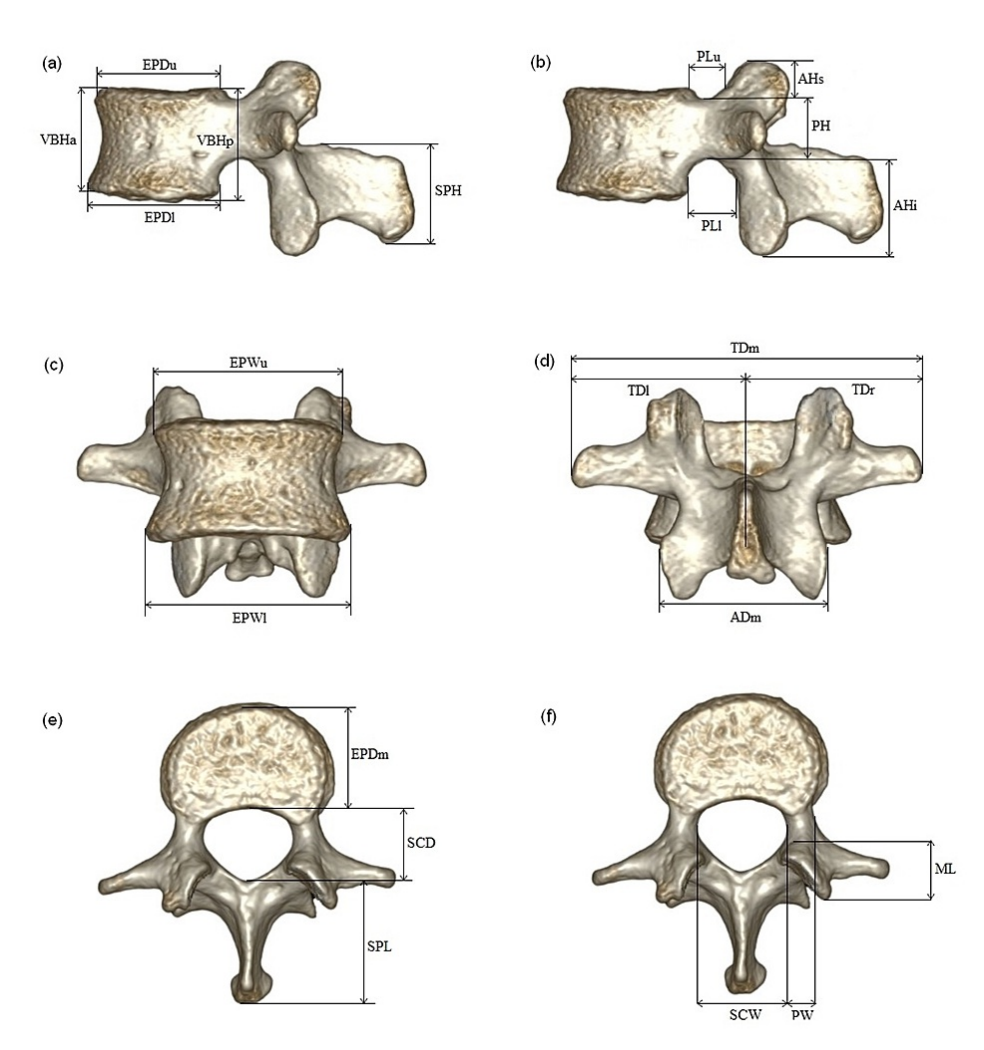
Three-dimensional views of L1 vertebra. (a) and (b) are lateral views; (c) and (d) are anterior and posterior views; (e) and (f) superior views. Adapted from Zheng et al. [11]. Permission for the re-print was obtained.

CT scans were performed using a 128-slice multi-detector computed tomography (MDCT) scanner (SOMATOM Definition Edge, Siemens Healthcare, Erlangen, Germany). Scan parameters were 100 kV, 186 mA, section thickness 1 mm, and reconstruction interval 0.6 mm. All scan data were transferred from the archive to a workstation (Aquarius workstation, TeraRecon, San Mateo, CA, USA) to create three-dimensional (3D) models of the L1 vertebra by post-processing procedures. Twenty-two linear measurements were taken in lateral, anterior, and superior views of 3D models of the L1 vertebra. The only left side of the parameter was measured for analyses except for transverse distance (TD). The measurements were performed by one radiologist with eleven years of experience in musculoskeletal radiology, one forensic pathologist with six years of experience in forensic anthropology. The measured parameters are shown in Figure 1 and listed in Table 1.

**Table 1:** Nomenclature and definition of the measurements. Derived from Zheng et al. [11].

Vertebral part	Symbol	Definition
Vertebral body	EPDm	Middle end-plate depth (Figure 1e)
	EPDu	Upper end-plate depth (Figure 1a)
	EPDl	Lower end-plate depth (Figure 1a)
	VBHa	Anterior height of vertebral body (Figure 1a)
	VBHp	Posterior height of vertebral body (Figure 1a)
	EPWu	Upper end-plate width (Figure 1c)
	EPWl	Lower end-plate width (Figure 1c)
Vertebral foramen pedicle	SCW	Spinal canal width (Figure 1f)
	SCD	Spinal canal depth (Figure 1e)
	PH	Pedicle height (Figure 1b)
	PW	Pedicle width (Figure 1f)
	PLu	Upper pedicle length (Figure 1b)
	PLl	Lower pedicle length (Figure 1b)
Articular process	AHs	Superior articular process height (Figure 1b)
	AHi	Inferior articular process height (Figure 1b)
	ADm	Maximum distance between articular processes (Figure 1d)
Mammillary process	ML	Mammillary process length (Figure 1f)
Transverse process	TD (r, l)	Transverse process distance (Figure 1d)
	TDm	Maximum distance between transverse processes (Figure 1d)
Spinous process	SPH	Spinous process height (Figure 1a)
	SPL	Spinous process length (Figure 1e)

The SPSS software (ver. 22; SPSS Inc., Chicago, IL) was used to assess the variables. After determining the mean and standard deviations for each variable, the differences between the genders were analyzed using Student’s t-test. P values less than 0.01 were considered statistically significant. Univariate and multivariate discriminant function analyses were applied to the measured parameters to determine predictive rates of sex. After all, measurements were completed by a radiologist, all parameters of 20 randomly selected people were measured again by the first observer and by a second observer. Intra- and interobserver errors were calculated using technical error of measurement (TEM), relative TEM (rTEM), and the coefficient of reliability (R) of the measurement, as suggested by Ulijaszek and Kerr [21].

## Results

In total, 241 subjects (121 females and 120 males) were enrolled in the study. The mean age of the total was 43 ± 16.4, the mean age of females was 44 ± 16.6, and the mean age of males was 41 ± 15.9. The mean and standard deviation values according to sex and P-values of the variables are shown in Table 2. All linear measurements were higher in males than females (Table 2). All parameters excluding SCD (Spinal canal depth), PLu (Upper pedicle length), PLI (Lower pedicle length), AHi (Inferior articular process height), and SPH (Spinous process height) showed statistically significant differences between sex (p < 0.01).

**Table 2:** Means, standard deviations and P-values for mean differences between females and males for all L1 vertebra variables.

Parameters	Sex	N	Mean	SD	P
						Middle end-plate depth (EPDm)	Female	120	3.055	0.324	0
Male	121	3.333	0.334								
Upper end-plate depth (EPDu)	Female	120	3.143	0.338	0
	Male	121	3.492	0.365	
Lower end-plate depth (EPDl)	Female	120	3.223	0.347	0
	Male	121	3.557	0.399	
Anterior height of vertebral body (VBHa)	Female	120	2.921	0.25	0.002
	Male	121	3.028	0.289	
Posterior height of vertebral body (VBHp)	Female	120	3.052	0.231	0
	Male	121	3.17	0.223	
Upper end-plate width (EPWu)	Female	120	4.049	0.376	0
	Male	121	4.45	0.422	
Lower end-plate width (EPWl)	Female	120	4.384	0.447	0
	Male	121	4.793	0.447	
Spinal canal width (SCW)	Female	120	2.056	0.27	0
	Male	121	2.197	0.27	
Spinal canal depth (SCD)	Female	120	1.653	0.257	0.64
	Male	121	1.67	0.287	
Pedicle height (PH)	Female	120	1.913	0.351	0.001
	Male	121	2.07	0.353	
Pedicle width (PW)	Female	120	0.89	0.226	0.001
	Male	121	0.991	0.236	
Upper pedicle length (PLu)	Female	120	0.825	0.212	0.845
	Male	121	0.82	0.223	
Lower pedicle length (PLi)	Female	120	1.207	0.265	0.771
	Male	121	1.218	0.28	
Superior articular process height (AHs)	Female	120	0.947	0.225	0.004
	Male	121	1.038	0.252	
Inferior articular process height (AHi)	Female	120	2.866	0.679	0.059
	Male	121	3.039	0.732	
Maximum distance between articular processes (Adm)	Female	120	2.78	0.488	0.002
	Male	121	2.989	0.55	
Mammillary process length (ML)	Female	120	2.275	0.421	0
	Male	121	2.505	0.489	
Right transverse process distance (TDr)	Female	120	3.652	0.421	0
	Male	121	3.999	0.452	
Left transverse process distance (TDl)	Female	120	3.494	0.425	0
	Male	121	3.795	0.433	
Maximum distance between transverse processes (TDm)	Female	120	7.008	0.656	0
	Male	121	7.558	0.659	
Spinous process height (SPH)	Female	120	2.641	0.276	0.874
	Male	121	2.634	0.379	
Spinous process length (SPL)	Female	120	2.848	0.33	0
	Male	121	3.119	0.428	

In calculating the correlations of the cases evaluated for intra-observer and inter-observer evaluation, for all the measured variables; correlations were found to be high in all measurement variables. R values ranged between 0.856-0.994 for intra-observer evaluation and ranged between 0.855-0.997 for the inter-observer evaluation. Technical measurement analysis results are presented in Table 3.

**Table 3:** Technical error measurement (TEM), relative TEM (rTEM) and R for intra- and inter-observer error for the variables included in the study.

Parameters	Intra-observer (n:20)			Inter-observer (n:20)		
	TEM	rTEM	R	TEM	rTEM	R
Middle end-plate depth (EPDm)	0.114	3.409	0.939	0.114	3.460	0.939
Upper end-plate depth (EPDu)	0.052	1.651	0.991	0.060	1.883	0.988
Lower end-plate depth (EPDl)	0.050	1.452	0.986	0.081	2.354	0.962
Anterior height of vertebral body (VBHa)	0.002	0.085	0.887	0.003	0.104	0.855
Posterior height of vertebral body (VBHp)	0.002	0.081	0.977	0.004	0.161	0.910
Upper end-plate width (EPWu)	0.039	0.904	0.993	0.092	2.152	0.962
Lower end-plate width (EPWl)	0.107	2.294	0.951	0.116	2.490	0.941
Spinal canal width (SCW)	0.094	4.436	0.856	0.060	2.844	0.936
Spinal canal depth (SCD)	0.065	3.921	0.938	0.052	3.212	0.952
Pedicle height (PH)	0.055	2.550	0.985	0.039	1.829	0.991
Pedicle width (PW)	0.085	8.452	0.893	0.043	4.360	0.971
Upper pedicle length (PLu)	0.056	7.115	0.949	0.037	4.766	0.977
Lower pedicle length (PLl)	0.061	4.754	0.965	0.085	6.611	0.948
Superior articular process height (AHs)	0.039	4.169	0.952	0.052	5.497	0.927
Inferior articular process height (AHi)	0.063	2.439	0.994	0.047	1.840	0.997
Maximum distance between articular processes (ADm)	0.067	2.234	0.977	0.115	3.905	0.925
Mammillary process length (ML)	0.147	6.278	0.896	0.068	2.932	0.977
Right transverse process distance (TDr)	0.173	4.552	0.910	0.060	1.558	0.989
Left transverse process distance (TDl)	0.047	1.300	0.992	0.112	3.086	0.961
Maximum distance between transverse processes (TDm)	0.157	2.170	0.953	0.093	1.281	0.983
Spinous process height (SPH)	0.002	0.102	0.963	0.004	0.204	0.888
Spinous process length (SPL)	0.129	4.342	0.867	0.071	2.386	0.952

Correct prediction rates of all single variables were calculated between 50.2%-70.5% to determine sex in the univariate discriminant analysis results of the variables measured for L1 vertebra. The highest rate of 70.5% was obtained for the EPWu (Upper end-plate width) and EPWI (Lower end-plate width) parameters. Univariate discrimination analysis results are presented in Table 4.

**Table 4:** Accuracy rates of univariate discriminant analysis.

Variable	Wilk’s Lambda	Correct Prediction Rates (%)		
		Female	Male	Overall
Middle end-plate depth (EPDm)	0.847	72.5	65.3	68.9
Upper end-plate depth (EPDu)	0.802	65	72.7	68.9
Lower end-plate depth (EPDl)	0.833	70.8	65.3	68.5
Anterior height of vertebral body (VBHa)	0.962	58.3	61.2	59.8
Posterior height of vertebral body (VBHp)	0.936	62.5	49.6	56.0
Upper end-plate width (EPWu)	0.798	74.2	65.3	70.5
Lower end-plate width (EPWl)	0.825	74.2	66.9	70.5
Spinal canal width (SCW)	0.936	65	54.5	59.8
Spinal canal depth (SCD)	0.999	58.3	44.6	51.5
Pedicle height (PH)	0.952	62.5	61.2	61.8
Pedicle width (PW)	0.954	62.5	54.5	58.5
Upper pedicle length (PLu)	1	39.2	60.3	50.2
Lower pedicle length (PLl)	1	55	45.5	50.2
Superior articular process height (AHs)	0.966	61.7	47.9	54.8
Inferior articular process height (AHi)	0.985	49.2	52.9	51.0
Maximum distance between articular processes (ADm)	0.961	58.3	57.9	58.1
Mammillary process length (ML)	0.940	64.2	58.7	62.2
Right transverse process distance (TDr)	0.863	65.8	62.8	64.3
Left transverse process distance (TDl)	0.890	64.2	65.3	64.7
Maximum distance between transverse processes (TDm)	0.843	42	65.3	67.6
Spinous process height (SPH)	1	48.3	57.9	53.1
Spinous process length (SPL)	0.888	66.7	63.6	65.1

As a result of multivariate discriminant analysis for L1 vertebra, correct prediction rates were 73.9% for the vertebral body parameters, 66.8% for the vertebral foramen and pedicle parameters, and 69.7% for all vertebral process parameters. When all variables of L1 vertebra were included in the stepwise discriminant analysis, correct prediction rates were determined as 72.6%. Multivariate discriminant analysis results were presented in Table 5.

**Table 5:** Accuracy rates of multivariate discriminant analysis.

Variable	Wilk’s Lambda	Accuracy Rates (%)		
		Female	Male	Overall
Body*	.693	71.9	75.8	73.9
Upper end-plate depth (EPDu)				
Middle end-plate depth (EPDm)				
Posterior height of vertebral body (VBHp)				
Upper end-plate width (EPWu)				
Lower end-plate width (EPWl)				
Foramen and pedicle**	.847	63.6	70.0	66.8
Spinal canal width (SCW)				
Pedicle height (PH)				
Pedicle width (PW)				
Process***	.775	70.0	69.4	69.7
Spinous process length (SPH)				
Mammillary process length (ML)				
Maximum distance between transverse processes (TDm)				
All Parameters	0.985	75.8	69.4	72.6
Spinous process length (SPL)				
Mammillary process length (ML)				
Maximum distance between transverse processes (TDm)				
Spinal canal width (SCW)				
Upper end-plate width (EPWu)				
Upper end-plate depth (EPDu)				

## Discussion

In our study, a significant sexual dimorphism was observed in 17 of 22 metric measurements made on the L1 vertebra. Not more than an 80% accuracy rate was observed for sex estimation in any of the metric measurements made on the L1 vertebra. The accuracy rates of these 17 metric measurements in which sexual dimorphism was detected varied between 50.2% and 70.5%. EPWu and EPWI measurements had the highest sex predicting accuracy (70.5%).

In the study of Ostrofsky and Churchill, L1 vertebra BSDVD (body superior dorsoventral diameter) and BSTD (body superior transverse diameter) measurements were found to have an accuracy rate of more than 80% [12]. In the study by Zheng et al. the EPWu measurement alone had the highest accuracy and EPWu, EPDm (middle end-plate depth), EPWI, EPDI (lower end-plate depth), EPDu (upper end-plate depth) measurements were found to have an accuracy rate exceeding 80% [11]. In the study of Decker et al. it is stated that only EPWu measurement has an accuracy rate of more than 80% [14]. In our study, the metric measurements made on the vertebral body of the L1 vertebra were the most dimorphic measurements, and the results were compatible with previous studies in the literature. However, in our study, it was observed that the accuracy rates of measurements made on L1 vertebrae were lower accuracy compared to other studies.

In previous studies, it has been stated that sex estimation can be made with an accuracy rate exceeding 80% with equations created by discriminant function analysis from metric measurements made on L1 vertebra [11-12,14-15] (Table 6). However, in our study population, an accuracy rate of more than 80% could not be obtained in the equations created using metric measurements on the L1 vertebra. Nevertheless, in our study, 17 measurements that exhibited significant sexual dimorphism were selected for discriminant function analysis, and it was shown that the equation created with six variables could assign sex with an accuracy of 72.6%.

**Table 6:** Summary of L1 vertebra-based sex estimation studies.

Author	Populations	Number	Sample	Accuracy (%)
Zheng et al. [11]	Chinese	210	CT scan	88.6
Ostrofsky and Churchill [12]	South African Blacks	98	Dry Bone	87.1
Decker et al. [14]	US North American	154	CT scan	83.8
Azofra-Monge and Alemán Aguilera [15]	Spanish	94	Dry Bone	94.5
Present study	Turkish	241	CT scan	72.6

The vertebral body provides strength and support for two-thirds of the vertebral load and is more resistant to mechanical stresses and taphonomic changes with its strong cortical and dense inner trabecular bone structure [18]. Although the possibility of obtaining the entire vertebral column intact is inherently high, the spinous processes and transverse processes of the vertebrae are more affected by taphonomic changes and might be fragmented [6,18]. Therefore, it may be more difficult to measure especially on articular facets. In our study population, it was seen that sex estimation could be made with an accuracy of 73.9% with the equation created from the measurements made only on the vertebral body. It was seen that the accuracy rate of the equation, which was created using the measurements related to the vertebral processes, was 69.7% in the sex estimation. In previous studies, it is seen that the measurements related to the vertebral body are dominant in the equations created for sex estimation from the L1 vertebra [11-12,15]. In the study of Decker et al. it was stated that the measurements of the vertebral pedicles in the obtained discriminant function equation were more effective in assigning sex [14]. However, in our study, there was a significant decrease in the accuracy rate (66.8%) in the equation created using the measurements on the vertebral foramen and pedicles. In our study; the equation created by the measurements made on the L1 vertebral body having a higher accuracy rate compared to other parts of the vertebrae also provides an advantage in terms of the vertebral body being more resistant to taphonomic changes. We think that the data from our study will be useful in cases where L1 vertebrae cannot be obtained as a whole but can be obtained partially. However, in instances where the vertebral column cannot be obtained as a whole or fragmented part of the lumbar vertebrae can be obtained, it may be a problem to determine which vertebra is L1 or which fragments belong to the L1 vertebra [12].

In our study, it was seen that the equation created with six variables could predict sex with an accuracy of 75.8% for females, while the accuracy rate was 69.4% for males. This difference between males and females may be due to the fact that vertebral sizes vary more among males than in females. In our study, it is seen that the standard deviations in the measurements of males are generally larger than the standard deviations of females. A similar situation has also been reported in previous studies examining the lumbar vertebrae [12-13].

The use of different measurement methods in the studies may also lead to differences in the obtained accuracy rates. Different results can be obtained from studies on dry bones and studies using radiological imaging methods. Slice thickness in radiological imaging with CT may also cause differences between studies. When3D reconstruction of anatomical features or other fine details is desired, it is recommended to use a maximum slice thickness of 1.25 mm [22]. Slice thicknesses greater than 1.25 mm will cause a loss of fidelity in the 3D reconstruction of images obtained [22]. Although it is recommended to use the narrowest slice thickness possible, it is also stated that there is no significant difference between measurements made using 1.25 mm slice thickness and measurements made using 0.625 mm slice thickness [22]. New 3D methods, which are increasingly used, may be useful in demonstrating sexual dimorphism patterns that have not been demonstrated by traditional methods [3]. In addition, radiological methods are also important in terms of developing non-invasive and non-destructive approaches. Although a 3D model is used in the study of Zheng et al. it is stated that measurements are made on-screen images [11]. On the other hand, Decker et al. measured directly from the marker points on the 3D model [14]. In our study, measurements were made directly on the 3D model with a slice thickness of 1 mm. Making the measurements directly on the 3D model will be useful in terms of the obtained study data being a reference for measurements to be made on dry bones.

It has been reported in many studies that skeletal age estimation studies differ between populations [6,20,23]. It has also been reported in previous studies that vertebral sizes differ between populations [12,24], as well as an Abstract by Pastor RF (Sexual Dimorphism in Vertebral Dimensions at the T12/L1 Junction. Proceedings of the 57th Annual Meeting of the American Academy of Forensic Sciences, Denver, CO; 2005). Therefore, it is a requirement that discriminant function analyses usage in estimating sex from lumbar vertebrae are population specific. Ancestry, genetics, environmental effects, socio-economic status, secular changes affect the development of the skeletal system. These differences were not taken into account in our study population. Therefore, lower sex prediction rates may have been obtained in our study compared to other studies, or the L1 vertebra in the Turkish population may show less sexual dimorphism compared to other populations. It is necessary to evaluate sexual dimorphism in L1 vertebrae and to establish population-specific discriminant functions by conducting studies in different populations.

## Conclusions

As a result, in our study; with metric measurements made on 3D CT images of the L1 vertebra, it was shown that sex estimation can be made with an accuracy rate of 72.6% in the Turkish population. Our study is the first study in which L1 vertebrae are examined with the purpose of sex estimation in the Turkish population and we think that our data will be an important reference for sex estimation from L1 vertebra in the Turkish population.
